# *Clostridium perfringens* Enterotoxin: Action, Genetics, and Translational Applications

**DOI:** 10.3390/toxins8030073

**Published:** 2016-03-16

**Authors:** John C. Freedman, Archana Shrestha, Bruce A. McClane

**Affiliations:** Department of Microbiology and Molecular Genetics, University of Pittsburgh School of Medicine, Pittsburgh, PA 15219, USA; jcf63@pitt.edu (J.C.F.); ars139@pitt.edu (A.S.)

**Keywords:** *Clostridium perfringens*, enterotoxin, pore-forming toxin, sporulation, tight junctions, claudins

## Abstract

*Clostridium perfringens* enterotoxin (CPE) is responsible for causing the gastrointestinal symptoms of several *C. perfringens* food- and nonfood-borne human gastrointestinal diseases. The enterotoxin gene (*cpe*) is located on either the chromosome (for most *C. perfringens* type A food poisoning strains) or large conjugative plasmids (for the remaining type A food poisoning and most, if not all, other CPE-producing strains). In all CPE-positive strains, the *cpe* gene is strongly associated with insertion sequences that may help to assist its mobilization and spread. During disease, CPE is produced when *C. perfringens* sporulates in the intestines, a process involving several sporulation-specific alternative sigma factors. The action of CPE starts with its binding to claudin receptors to form a small complex; those small complexes then oligomerize to create a hexameric prepore on the membrane surface. Beta hairpin loops from the CPE molecules in the prepore assemble into a beta barrel that inserts into the membrane to form an active pore that enhances calcium influx, causing cell death. This cell death results in intestinal damage that causes fluid and electrolyte loss. CPE is now being explored for translational applications including cancer therapy/diagnosis, drug delivery, and vaccination.

## 1. Introduction

*Clostridium perfringens* is a preeminent pathogen of humans and livestock, causing both histotoxic diseases and illnesses originating in the intestines, namely enteritis or enterotoxemia (where toxins produced in the intestine are absorbed into the circulation and then damage organs such as the brain) [1]. The virulence of this Gram-positive, spore-forming anaerobe is largely attributable to its ability to produce at least 17 different toxins [2]. However, there is considerable variability in the toxin armamentarium of different *C. perfringens* strains, which provides the basis for a toxinotyping classification system that divides *C. perfringens* isolates into five types (A–E) depending upon their ability to produce alpha, beta, epsilon, and iota toxin [2].

About 5% of all *C. perfringens* isolates produce a toxin named *C. perfringens* enterotoxin (CPE) [3]. Most CPE-positive strains classify as type A, although types C and D strains producing this enterotoxin are also fairly common [4]. The primary amino acid sequence of the CPE protein made by CPE-positive types A, C, and D strains is virtually identical [4]. It had been thought that type E strains only carry silent *cpe* sequences [5], but a recent study identified a few type E strains that produce a variant CPE [6]. To date, there are no reliable reports of CPE production by type B strains.

## 2. The Importance of CPE in Gastrointestinal (GI) Disease

**Figure 1:**
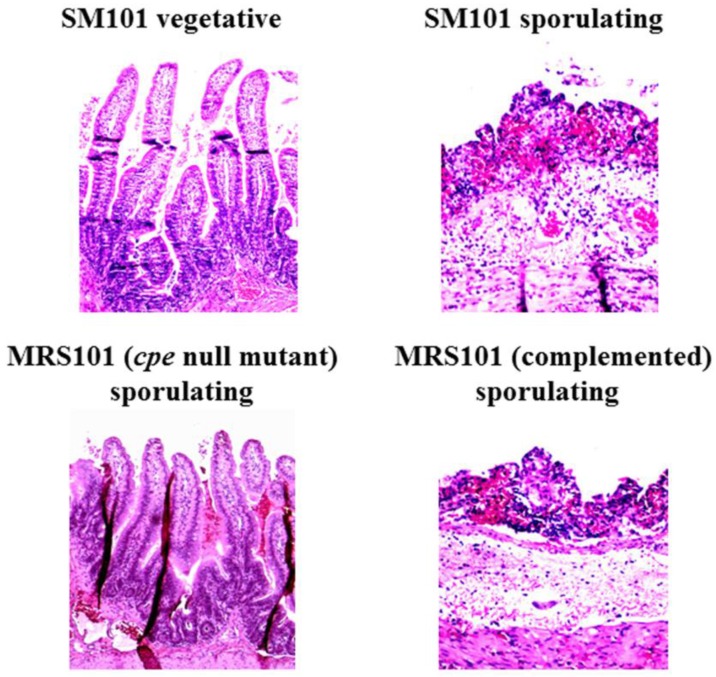
Histologic damage induced by lysates of CPE-positive type A strain SM101, a transformable food poisoning strain derivative. Shown are hematoxylin and eosin-stained tissue sections from rabbit ileal loops treated with concentrated vegetative culture lysates (FTG) or concentrated Duncan-Strong (DS) sporulating culture lysates of wild-type SM101, MRS101 (a *cpe* null mutant of SM101) or a complementing strain where the *cpe* gene has been transformed back into MRS101. Note the complete absence of damage using lysates of SM101 vegetative cultures or DS cultures of the MRS101 *cpe* null mutant. However, extensive necrosis, villus damage, and epithelial desquamation were observed using lysates of the DS culture of wild-type SM101 or the complementing strain. Samples shown are at 250 × magnification. Reproduced with permission from [10].

*Clostridium perfringens* type A food poisoning ranks as the second most common foodborne illness in most developed countries [7]. For example, there are approximately one million cases of this food poisoning each year in the United States, causing annual economic losses of ~$400 million [8,9]. Compelling epidemiologic and experimental evidence indicates that CPE is the toxin responsible for the diarrhea and abdominal cramping symptoms that are characteristic of type A food poisoning [7]. The most persuasive laboratory evidence linking CPE to this foodborne illness was provided by a study [10] reporting that inactivation of the *cpe* gene in a human food poisoning strain renders that strain avirulent in animal models of enteric disease, with this attenuation reversible by complementation to restore CPE production (Figure 1). One example of the abundant epidemiologic evidence supporting a role for CPE in *C. perfringens* type A food poisoning is the direct detection of CPE in feces from most patients with this foodborne illness [7].

It deserves brief mention that two studies recently linked CPE-negative type A strains to some food poisoning cases in Japan [11,12]. However, the responsible toxin made by those strains resembles iota toxin, so those cases might better be considered as type E human food poisoning. The prevalence of these new type E-like strains in human food poisoning requires further study, but it seems clear that CPE-positive type A strains are responsible for the great majority of human food poisoning cases caused by *C. perfringens*.

In the past decade two unusually severe outbreaks of *C. perfringens* type A food poisoning occurred that resulted in the deaths of several relatively young and healthy people [13,14]. These outbreaks occurred in psychiatric hospitals and involved patients receiving psychoactive drugs, which often have constipation or fecal impaction side-effects. It is believed that those side-effects interfered with the development of the diarrhea typical of most *C. perfringens* type A food poisoning cases. Reducing diarrhea would cause prolonged contact between the intestines and toxins like CPE, which may have facilitated the absorption of CPE into the circulation so it could damage non-intestinal organs. This hypothesis received direct experimental support from mouse studies demonstrating absorption of CPE from static small intestinal loops [15]. The absorbed CPE then bound to the liver and kidneys, producing an enterotoxemia that resulted in organ damage causing hyperpotassemia, which induced death by cardiac arrest in mice.

In addition to *C. perfringens* type A food poisoning, CPE-positive type A strains also cause several non-food-borne human gastrointestinal diseases, including about 5%–10% of all cases of antibiotic-associated diarrhea [16]. Animal model studies [10] with *cpe* null mutants and complementing strains of a CPE-positive non-food-borne human GI disease strain showed that CPE production is required for the enteropathogenicity of these strains. Other evidence supporting the importance of CPE for some cases of non-food-borne human GI disease cases includes the direct detection of this toxin in feces from ~5% to 10% of people with these diseases [16].

CPE may also contribute to some cases of human enteritis necroticans (EN) caused by type C strains. EN is a foodborne illness that is associated with low intestinal trypsin levels due to disease and/or diet [2]. While beta toxin is clearly essential for the pathogenesis of EN, some type C strains causing this disease also produce CPE [17]. Recent animal model studies [18] using toxin mutants and purified toxins demonstrated synergistic effects for CPE and beta toxin, suggesting that (when present together with beta toxin in the intestines) CPE can contribute to the pathogenesis of enteritis necroticans. Whether CPE, when produced, contributes to type D disease is still unknown.

## 3. Introduction to the CPE Protein

Cloning of the *cpe* gene in 1993 [19] revealed that the CPE protein produced by a type A food poisoning strain is a single polypeptide containing 319 amino acids with a unique primary sequence. Later *cpe* nucleotide sequencing studies showed that the CPE produced by other CPE-positive type A strains shares this same sequence [20], as does the CPE made by some type C and D strains of *C. perfringens* [4]. Interestingly, a few type E strains produce a variant CPE with 10 amino acid differences from the classical CPE made by types A, C, and D strains [6]. The phenotypic consequences of those amino acid variations for toxicity are unknown at present.

There has been intensive analysis of the structure *vs.* function relationship for the CPE protein. Early biochemical and cloning studies [21,22] determined that the *C*-terminal half of CPE lacks cytotoxic activity, although it binds strongly to receptors. Synthetic peptide and subcloning approaches then mapped strong receptor binding activity to the last 30 amino acids of the toxin [23]. Several tyrosine residues in this extreme *C*-terminal region were later shown to be important for receptor binding [24,25]. More recent studies identified additional residues in the *C*-terminal half of CPE that also contribute to receptor binding [26].

The first 44 amino acids of CPE are not necessary for cytotoxicity [27]. In fact, removing these amino acids by deletion mutagenesis actually increases CPE cytotoxic activity by ~2–3 fold. This effect likely has significance for CPE-mediated disease since the intestinal proteases trypsin and chymotrypsin, which remove ~25 or 37 amino acids (respectively) from the *N*-terminus of native CPE, also produce an activated toxin [28]. Note that these *N*-terminal sequences are not a signal peptide since CPE is not secreted but instead accumulates inside a sporulating cell until that mother cell lyses to release its spore (further discussion later).

Deletion of amino acids beyond amino acid 44 quickly abolishes CPE action [27]. Site-directed mutagenesis studies [29] then identified several amino acids, particularly residues D48 and I51, in the *N*-terminal half of CPE as being extremely important for cytotoxicity because these amino acid residues are needed for CPE oligomerization and pore formation.

However, oligomerization is not the only function mediated by the *N*-terminal half of CPE. *N*-terminal CPE residues 80–106 (a region referred to as TM1) consist of alternating hydrophilic and hydrophobic amino acids, which resemble the β-hairpin loops that are known to mediate membrane insertion and pore formation for other bacterial pore-forming toxins [30]. Site-directed mutagenesis has confirmed that this TM1 region is important for both CPE membrane insertion and pore formation [30].

In 2008, Van Itallie *et al.* [31] solved the structure of the *C*-terminal half of CPE (also referred to as C-CPE). This structural analysis showed that the C-CPE domain is a nine-strand β-sandwich with some resemblance to the receptor-binding domain of several other pore forming toxins. Amino acid residues implicated in receptor binding form a pocket on the surface of C-CPE.

**Figure 2:**
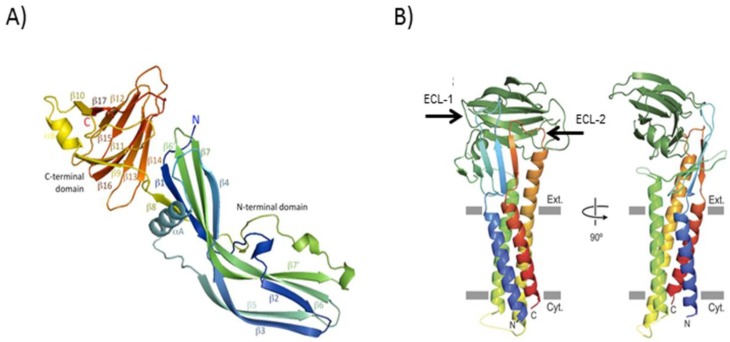
Structure of the CPE monomer (**left**) and C-CPE bound to a claudin receptor (**right**). Left panel (**A**) shows the two domain structure of the CPE monomer, consisting of the *C*-terminal CPE binding domain (C-CPE, **yellow-red**) and the *N*-terminal oligomerization/membrane insertion domain. Reproduced with permission from [32]. Right panel (**B**) shows the C-CPE binding domain bound to a claudin receptor, which consists of four transmembrane domains, two extracellular loops and a short *C*-terminal tail (not shown). Note that C-CPE interacts with both claudin extracellular loops but the second extracellular loop distinguishes claudins capable of binding CPE from those unable to bind the toxin. Reproduced with permission from [34].

In 2010, the structure of the entire CPE protein was independently solved by two groups (Figure 2A). Those studies [32,33] determined that CPE is a two-domain protein consisting of the *C*-terminal receptor binding domain described above and a distinct *N*-terminal domain that is involved in oligomerization and pore formation. The extreme *N*-terminus (residues 1–34) of CPE has no interpretable density, which suggests disorder and may help to explain why these sequences, when present, partially reduce CPE activity, *i.e.*, they may sterically hinder oligomerization. The TM1 region mediating membrane insertion and pore formation largely corresponds to an alpha helix located in the *N*-terminal domain of CPE. This alpha helix likely unfolds into a *β*-hairpin loop during membrane penetration and pore formation by the CH-1 complex [32,33].

Resolution of the CPE structure also assigned this toxin to the aerolysin pore-forming toxin family, which also includes (among other toxins) *C. perfringens* epsilon toxin [35]. Interestingly, CPE is unique among members of this pore-forming toxin family since the region involved in β-hairpin formation is an alpha helix when CPE is in the soluble monomeric form.

## 4. *cpe* Genetics

Approximately 5% of global isolates produce CPE [3], which (as mentioned earlier) can be encoded for by types A, C, D, and E strains of *C. perfringens* [4,6]. The enterotoxin gene (*cpe*) can be located on either the chromosome or on plasmids [2]; no isolate has yet been found that carries both a chromosomal and plasmid-borne *cpe* gene.

Most (~70%) type A food poisoning strains carry a chromosomal copy of the *cpe* gene [7]. This chromosomal *cpe* gene is proximally associated with insertion sequences, including a IS*1469* sequence located 1.3 kb upstream of the *cpe,* gene and two IS*1470* sequences, one of which is present 3 kb upstream of the *cp*e gene and the other located 1.2 kb downstream of the *cpe* gene [36,37,38]. It has been proposed, though not yet proven, that this chromosomal *cpe* gene with its two flanking IS*1470* sequences corresponds to a transposon [36]. Consistent with that possibility, PCR has detected circular forms carrying the *cpe* gene in type A chromosomal *cpe* strain NCTC8239 that may represent transposition intermediates [39].

In addition to carrying this putative transposon, chromosomal *cpe* type A strains are phylogenetically distinct from other *C. perfringens.* Helping to explain their strong association with food poisoning*,* most chromosomal *cpe* type A strains produce a variant small acid soluble protein that binds tightly to spore DNA, which provides these chromosomal *cpe* strain spores with much stronger resistance against food environment stresses, such as heating, than exhibited by spores of other *C. perfringens* strains [40,41,42].

The remaining ~30% of type A food poisoning strains, and virtually all CPE-positive type A non-food-borne human GI disease *C. perfringens* strains, carry their *cpe* gene on plasmids of ~70–75 kb [2,43]. In type A strains, all known *cpe*-positive plasmids belong to either the pCPF5603 or pCPF4969 plasmid families [44]. Both *cpe* plasmid families in type A strains are thought to have derived from a common precursor plasmid (a pCP13-like plasmid), but the pCPF5603 plasmid family now also carries the gene encoding beta2 toxin and a cluster of putative metabolic genes, while the pCPF4969 plasmid family now also carries a VirS/VirR-like two-component regulatory system and a putative bacteriocin [44]. Like the chromosomal *cpe* gene, the plasmid-borne *cpe* gene in type A strains is closely associated with insertion sequences, which could mobilize this toxin gene. In both *cpe* plasmid families found in type A strains, the *cpe* gene is flanked by a 5′ IS*1469* insertion sequence. However, the *cpe* gene is usually flanked 3′ by IS*1470* in pCPF4969 family plasmids but by IS*1151* in pCPF5603 family plasmids [44]. In addition to the close association between insertion sequences and the plasmid-borne *cpe* gene of type A strains, these *cpe* genes may be further mobilized by their presence on conjugative plasmids [45]. pCPF4969 family plasmids have been shown to conjugatively transfer among *C. perfringens* strains, probably due to their carriage of similar *tcp* sequences demonstrated to mediate conjugative transfer of other *C. perfringens* plasmids [45]; pCPF5603 family plasmids are likely to also be conjugative since they carry similar *tcp* sequences [44].

In types C, D, and E strains, the *cpe* gene is also located on plasmids, but these are usually larger in size than the *cpe* plasmids of type A strains [46]. In type C strains, the *cpe* plasmid is usually either ~85 kb or ~110 kb [4]. Interestingly, the plasmid *cpe* locus in some type C strains closely resembles that found in pCPF5603 and has both a 5′ IS*1469* insertion sequence and a 3′ IS*1151* insertion sequence. However, the plasmid *cpe* locus in other type C strains more closely resembles the chromosomal *cpe* locus of type A strains with a 3′ IS*1470* sequence, along with an additional IS*1470*-like sequence, that is located upstream of the *cpe* gene but downstream of the 5′ IS*1469* element [47]. In type D strains, the *cpe* gene can be found on ~75, 85, or 110 kb plasmids. The sequenced *cpe* loci in type D strains have a unique genetic organization, with two putative upstream transposase genes similar to Tn*1546* and downstream sequences similar to that found in the *cpe* plasmid in type A strain F4969; however the type D *cpe* locus lacks an IS*1470*-like insertion sequence [4]. Finally, type E strains of *C. perfringens* commonly carry plasmids with the *cpe* gene or silent *cpe* sequences*.* When carrying silent copies of *cpe,* those plasmids resemble pCPF5603, but are much larger (~97 or 135 kb) and carry iota toxin genes that have apparently inserted upstream of their *cpe* gene, thereby eliminating *cpe* expression [48]. In contrast, a recent study discovered type E strains with an ~65 kb pCPF4969-like plasmid that carries an expressed copy of *cpe* [6].

Taken together, the above findings clearly indicate that the *cpe* gene is present in a diverse range of *C. perfringens* strains, where it can reside either on the chromosome (in most food poisoning isolates) or on a number of different, but often related, conjugative plasmids. In addition, the *cpe* gene is often associated with flanking insertion sequences and/or transposases [4,6,36,38,44,48]. This association of the *cpe* gene with mobile genetic elements likely assists its mobilization and transfer, thereby impacting the evolution of virulence in many intestinal disease-causing strains of *C. perfringens.*

## 5. CPE Production and Regulation

Regardless of whether it is chromosomally- or plasmid-encoded, the *cpe* gene is only expressed during sporulation [2,7], when *C. perfringens* undergoes asymmetrical cell division under nutritionally depleted conditions. This process results in the production of a mature spore that is dormant and can withstand harsh environmental conditions, such as cooking, that are encountered in the food environment. *C. perfringens* food poisoning often occurs when the exceptionally-resistant spores of type A chromosomal *cpe* strains survive improper cooking and then germinate into vegetatively growing cells. Those bacteria grow to high numbers in the contaminated food, which is then consumed. If large numbers of vegetative cells are ingested, some survive the acidity of the stomach and then passage into the intestine, where they initially expand in numbers. However, once present in the intestine, these vegetative cells soon commit to sporulation and begin to produce CPE. At the completion of sporulation, the mother cell lyses, which releases CPE into the intestinal lumen so it can act, as described later [7].

During non-food-borne CPE-mediated human gastrointestinal disease*,* spores are thought to be the actual infectious agent [16], followed by repeated cycles of spore germination and sporulation (along with CPE production) in the intestine [16]. This cycling may be due to greater colonization ability by non-food-borne GI disease strains [49] and likely explains the longer duration of these illnesses compared to *C. perfringens* type A food poisoning. The presence of the *cpe* gene on conjugative plasmids in the non-food-borne GI disease strains may contribute to these illnesses by converting colonization-proficient, but naturally CPE-negative, normal flora *C. perfringens* strains to intestinal virulence [45].

Similarly as sporulation in other *Clostridium* spp and in *Bacillus* spp., *C. perfringens* sporulation involves a hierarchical cascade of regulation [50,51]. During the *in vivo* sporulation of *C. perfringens*, an unknown signal, possibly the presence of inorganic phosphate or the presence of bile salts, leads to the activation of the master sporulation regulator Spo0A [52,53]. Studies in other *Bacillus and Clostridium* spp. also demonstrated that Spo0A is a response regulator protein whose activity is dependent on phosphorylation; the phosphorylated Spo0A then binds to regions upstream of genes required for sporulation. Once bound, phosphorylated Spo0A activates the expression of these sporulation genes [54]. Using knock-out mutants, it has been established that Spo0A is also required for *C. perfringens* to sporulate and produce CPE [55].

**Figure 3:**
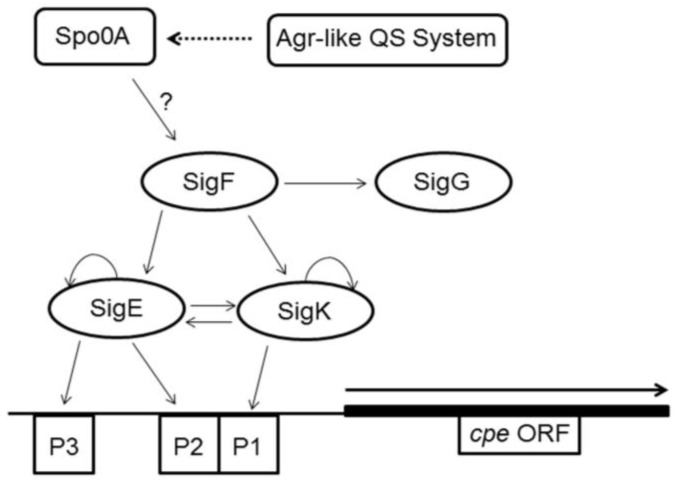
Model for regulation of CPE production. This updated model for CPE regulation integrates result regarding the roles of the master regulator of sporulation Spo0A [54], the sporulation-specific sigma factors [50,51], and the Agr-like quorum sensing system [57] in the regulation of CPE production. Note that the precise mechanisms of regulation of CPE production by Spo0A and the Agr system remain unknown and thus indirect interactions are depicted with broken arrows or arrows and question marks. All regulatory factors shown are required for sporulation and all but SigG are also required for CPE production. CcpA (not shown) also controls CPE production and sporulation through an unknown pathway [58]. Adapted from [50].

Among the target genes that are likely to be activated by phosphorylated Spo0A in *C. perfringens* is the gene encoding SigF [50,54]. SigF is an alternative sigma factor that is required for *C. perfringens* sporulation and CPE production [50]. Furthermore, using a *C. perfringens sigF* null strain, it was shown that SigF regulates production of downstream alternative sporulation-specific sigma factors, *i.e.*, SigG, SigK, and SigE, each of which is required for the completion of sporulation in *C. perfringens* [50]. However, besides SigF (which regulates production of the other three sigma factors), SigK, and SigE (but not SigG) are required for CPE production [51]. This is explainable by the presence of three promoters upstream of the *cpe* gene (whether chromosomal or plasmid) in type A strains that regulate the expression of *cpe* [56]. The first of these promoters (P1) contains sequences similar to previously described SigK recognition sequences, while the second and third promoters (P2 and P3) demonstrate similarity to recognition sequences for SigE [56]. Taken together, the current model proposes that phosphorylated Spo0A activates the transcription of SigF, which then activates the transcription of SigG, SigK, and SigE, all of which are required for sporulation, with the latter two also required for CPE production (Figure 3).

In addition to phosphorylated Spo0A and the sporulation-specific alternative sigma factors, several other global regulators have been shown to play a role in sporulation and production of CPE by *C. perfringens* [57,58,59]. Among those regulators are catabolite control protein A (CcpA), a global regulatory protein found in many Gram-positive bacteria that directly or indirectly regulates the expression of many genes involved in carbon and nitrogen utilization [58,60]. When the *ccpA* gene was disrupted by homologous recombination in type A *C. perfringens* strain SM101, both sporulation and CPE production were drastically reduced [58]. However, the mechanism involving this CcpA-dependent regulation of sporulation and CPE production remains unknown. In addition to CcpA, the Agr-like quorum sensing system of *C. perfringens* also regulates sporulation and CPE production by type A strain F5603 [57]. Specifically, this quorum sensing system positively controls the production of Spo0A and, thus, SigF, SigE, CPE and sporulation [57]. A more recent study demonstrated that the small RNA *virX* negatively regulates sporulation and CPE production, as demonstrated by an increase in SigE, SigF, and SigK expression and CPE production during sporulation [59]. Taken together, the tightly controlled regulation and production of CPE during *C. perfringens* sporulation is clearly dependent upon several global regulators and alternative sporulation sigma factors that act in a hierarchical manner.

## 6. The Cellular Action of CPE

Claudins form the backbone [61] of tight junctions (TJs), which are located at apical cell-cell contact regions in epithelial and endothelial cells. Claudins can form fibrils that play important roles in modulating the structure and function of mammalian TJs [61,62,63]. The claudin family consists of 27 different proteins, which typically are 20–27 kDa in size. Claudins are comprised of four transmembrane domains, a short *C*-terminal cytoplasmic tail and two extracellular loops (named ECL-1 and ECL-2) [64].

Certain members of the claudin tight-junction protein family are functional receptors for CPE binding to host cells. Specifically, claudins 3, 4, 6, 8, and 14 are proven CPE receptors [65,66,67,68]. However, not all 27 members of the claudin family are CPE-receptors as claudins 1, 2, 5, and 10 do not bind CPE at pathophysiologically relevant toxin concentrations [66,67,68,69].

Studies using chimeric claudins showed that the ECL-2 region is critical for a claudin to bind CPE [66]. Peptide mapping studies identified a pentapeptide sequence within the ECL-2 region that is important for CPE binding [70]. Site-directed mutagenesis showed that an Asn residue located in this pentapeptide is a critical yes/no determinant for CPE binding, with adjacent residues modulating the affinity of CPE binding [71] (note that some claudin receptors, like claudin 4, bind CPE very tightly, while other receptors, such as claudin 8, bind the toxin with less affinity [67]).

Given those findings documenting the importance of CPE:ECL-2 interactions, it was interesting when recent structural analyses (Figure 2B) revealed that both the ECL-1 and ECL-2 regions of claudin receptors interact with CPE and that the interactions between ECL-1 and the toxin are also necessary for binding [34]. The emerging picture is that all claudins can carry out the necessary interactions between ECL-1 and the CPE binding domain, as supported by studies [65] with chimeric claudins showing that the *N*-terminal half (containing the ECL-1 region) can be interchanged between receptor and non-receptor claudins without affecting CPE binding. However, only the receptor claudins possess a suitable ECL-2 region for CPE binding [66,71].

**Figure 4:**
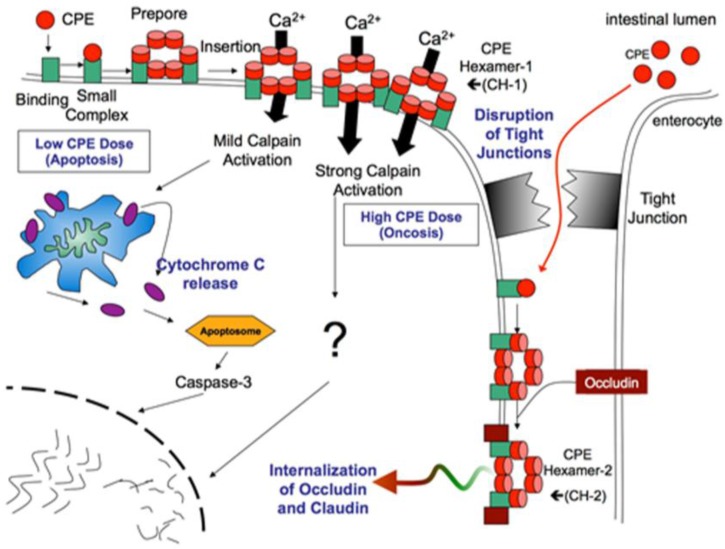
The mechanism of action of *C. perfringens* enterotoxin. CPE first binds to claudin receptors (**green** boxes) present on the apical surface of host cells and forms a small complex. Several (approximately six) small complexes, which also contain non-receptor claudin, not shown), interact to promote CPE oligomerization and form a prepore on the plasma membrane surface. β-hairpins in the CPE molecules of the prepore then form a β-barrel that inserts into membranes to form an active pore named CH-1. Low-dose CPE treatment causes a limited calcium influx and some cytoplasmic calpain activation to cause caspase-3 mediated apoptosis. High dose CPE treatment causes a strong calcium influx cytoplasmic calpain activation to induce cell death via oncosis. Morphologic damage to the cells exposes the basolateral surface of cells, which allows CPE access to other receptors to form additional CH-1 complex. Further, this basolateral surface exposure allows for the formation of a second large complex named CH-2, which triggers internalization of CPE-non receptor occludin and claudin into the cytoplasm. Modified with permission from [74].

Binding of CPE to claudin receptors results in the formation of a ∼90 kDa, “small complex” that contains CPE and both receptor and non-receptor claudins (Figure 4) [72,73]. This small complex is, by itself, insufficient to trigger cytotoxicity; instead, several small CPE complexes interact to promote CPE oligomerization to form a prepore on the plasma membrane surface [74]. This oligomerization results in the formation of a ~450 kDa CPE “large complex” named CH-1, that contains a CPE hexamer and both receptor and non-receptor claudins [72].

β-hairpin loops from CPE in the CH-1 complex assemble into a β-barrel that quickly inserts into membranes to form a pore [30,75], causing plasma membrane permeability alterations in sensitive mammalian cells [76,77,78,79]. The CPE pore is cation permeating [76] and permits a calcium influx that is important if not essential for CPE-induced cell death [78,79,80,81,82].

Low CPE doses result in formation of a low number of pores, causing a modest calcium influx [81,82]. Via a process involving calmodulin and a modest calpain activation, this limited calcium influx triggers a caspase-3 mediated death of the CPE-treated cell with all the hallmarks (e.g., cytochrome c release) of classical apoptosis. In contrast, higher CPE doses cause formation of many pores, producing a massive calcium influx [83]. Via a process involving calmodulin and a very strong calpain activation, this overwhelming calcium influx results in a form of necrotic cell death known as oncosis [83].

With extended time, morphological damage develops in CPE-treated cells, which exposes the basolateral cell surface. This allows additional binding of the toxin such that the toxin now forms an even larger (~600 kDa) complex named CH-2, which contains claudins and occludin [72,84], a structural component of epithelial TJs. The contribution of CH-2 to cytotoxicity is unclear but it may help to explain the observed internalization of occludin (and claudins) into CPE-treated cells.

## 7. CPE *in vivo* Effects

**Figure 5:**
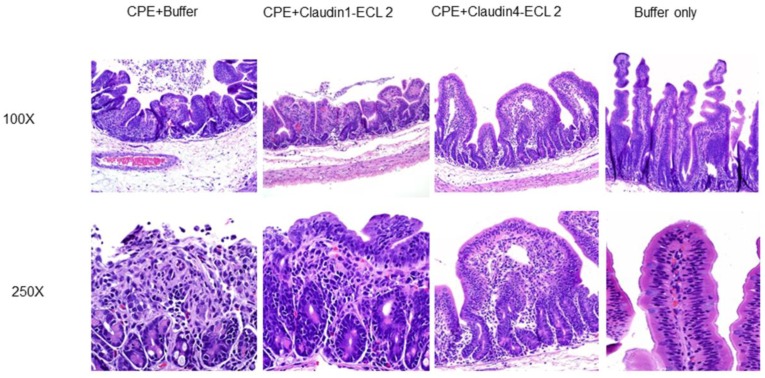
A synthetic peptide corresponding to the ECL-2 region of the claudin 4 CPE receptor can act as a decoy receptor and interfere with CPE action *in vivo*. Hematoxylin and Eosin-stained histology (100×, **top**; 400×, **bottom**) shown for: Control (loops treated with buffer alone); CPE + buffer (loops) treated with CPE and buffer but no synthetic peptide); CPE + Cldn-1 ECL-2 (loops treated with CPE and a synthetic peptide with the ECL-2 sequence of non-CPE receptor claudin 1); CPE + Cldn-4 ECL-2 (loops treated with CPE and a synthetic peptide with the ECL-2 sequence of CPE receptor claudin 4). All results are shown after a 1-h incubation. Modified with permission from [90].

CPE causes histologic damage in the small intestine of all tested mammalian species, presumably including humans since the toxin similarly damages human small intestinal tissue *ex vivo* [15,85,86,87,88]. CPE-induced histologic damage includes severe villus shortening, along with epithelial necrosis and desquamation [15,86,87,88]. Animal model studies support a role for CPE-induced cytotoxicity in the development of this histologic damage, *i.e.*, noncytotoxic, but binding-capable, CPE derivatives fail to cause the development of histologic damage in rabbit small intestines [89]. Further linking the cellular action of CPE with the toxin’s intestinal effects are observations that CPE first damages villi tips in rabbit small intestine, which have an abundant presence of the CPE receptor claudin 4 [89] (Figure 5).

CPE-induced intestinal damage appears to be an important, if not essential, contributor to CPE-induced fluid and electrolyte secretion. For example, in rabbit small intestinal loops, a close temporal association exists between the onset of CPE-induced histologic damage and the start of fluid and electrolyte loss [87]. Furthermore, fluid and electrolyte losses only develop in the small intestines of animal models treated with CPE doses capable of causing histologic damage [86].

For many years it was believed that CPE had no effect on the colon, despite the ability of this organ to bind high levels of the enterotoxin due to the presence of receptor claudins [88]. However, a recent study demonstrated that CPE does induce histologic damage and fluid/electrolyte losses in the rabbit colon [91]. This result may help to explain the necrotizing colitis observed in a severe *C. perfringens* type A food poisoning outbreak that occurred in patients of an Oklahoma psychiatric facility [13].

As mentioned earlier, studies with CPE-challenged mouse small intestinal loops directly demonstrated that CPE can be absorbed through the intestines [15]. This appearance of the enterotoxin in the circulation leads to CPE binding to nonintestinal organs such as the liver and kidneys. Those interactions lead to a lethal enterotoxemia, largely due to a hyperpotassemia that likely induces cardiac arrest in these mice [15]. It has been proposed that CPE-induced enterotoxemia could explain the severity of the previously mentioned *C. perfringens* type A food poisoning outbreaks in psychiatric hospitals [13,14]. Those outbreaks involved the deaths of several relatively young and healthy people with preexisting psychotropic drug-induced constipation or fecal impaction side-effects, which likely delayed diarrhea and prolonged contact of CPE with the intestines, fostering toxin absorption [14].

## 8. Potential CPE Vaccine

A series of defined CPE fragments were reacted with a panel of CPE-specific monoclonal antibodies (MAbs) in epitope mapping studies [28]. Those analyses identified at least four regions, scattered throughout the enterotoxin protein, that are involved in epitope presentation. A linear epitope recognized by one anti-CPE MAb, *i.e.*, MAb 3C9, maps to the extreme *C*-terminal 30 amino acid region of the enterotoxin. The *C*-terminal region of CPE is involved in binding of the enterotoxin to claudins (see Section 3), so the reactivity of MAb 3C9 with an epitope located in this region is fully consistent with observations that this monoclonal is a neutralizing antibody that blocks CPE receptor binding [92].

Since *C*-terminal CPE fragments are not cytotoxic [22,23], the presence of a neutralizing epitope in those fragments suggested they might be potential CPE vaccine candidates. This possibility was tested by conjugating a thyroglobulin carrier with a synthetic peptide corresponding to the *C*-terminal 30 amino acids of CPE [93]. When mice received an intravenous injection of this conjugate, they mounted a strong CPE-neutralizing antibody response. While this conjugate has vaccine potential, it has not yet been tested for the ability to stimulate a mucosal IgA response, as would be necessary for intestinal protection against CPE-mediated disease.

## 9. Translational Applications of CPE

Many human normal or cancer cells express claudins that can function as CPE receptors. Exploiting that observation, the CPE protein (or non-cytotoxic CPE derivatives such as C-CPE) are now being actively explored for a variety of translational applications.

It has become well established that cancer cells, particularly prostate, breast, pancreatic, and ovarian cancer cells, upregulate their expression of claudin CPE receptors [94]. In response, considerable effort has been made towards developing CPE-based approaches for cancer therapy and diagnosis [94]. As an example of animal model studies supporting this application, CPE was shown to suppress pancreatic tumor growth in mice [95]. Alternatively, a novel therapeutic has been developed that consist of C-CPE and active agents such as the protein synthesis inhibitory region of *Pseudomonas* exotoxin A; those chimeric proteins were demonstrated to selectively damage tumor cells [96]. Another approach used C-CPE to sensitive ovarian cancer cells to treatment with conventional cancer therapeutics, including taxol and carboplatin [97]. In addition to treatment, labeled C-CPE-based derivatives have been used for imaging and detecting tumors [98]. For example, micrometastatic human ovarian cancer tumor implants in mice have been visualized using fluorescently-labeled C-CPE [98].

For mucosal immunizations the important first step is delivering the desired antigen to mucosal-associated lymphoid tissue (MALT). MALT contains multiple cell types, including M cells, that take up and present antigen to underlying antigen-presenting cells for processing. M cells express high levels of claudin 4, a high-affinity CPE receptor, so fusion proteins containing the C-CPE claudin binding domain represent attractive agents for enhancing mucosal immunizations [99]. An example of several studies exploring this immunization approach used C-CPE fused with pneumococcal surface protein A (PspA). When administered intranasally, this vaccine induced protective immunity against challenge with the important bacterial pathogen *Streptococcus pneumoniae* [99]. Similarly, a peptide fused to the *C*-terminal 30 amino acids of CPE has been tested as part of a conjugate vaccine to protect mice from Coxsackie virus B3 infection [100].

A final translational use of CPE derivatives has been to enhance drug absorption across mucosal tissue [101]. The rationale for this approach is based upon observations that C-CPE can open tight junctions, thus increasing drug paracellular permeability. In fact, observations indicate that C-CPE has ~400-fold more activity than clinically used enhancers of drug delivery [101]. Current efforts are developing even more potent and specific C-CPE-based drug delivery enhancers by mutagenesis approaches [24]. For example, engineered C-CPE variants recognizing only one or a few specific claudins (e.g., claudin 4) can enhance insulin delivery across human nasal epithelial cells [102].

## 10. Concluding Remarks

The increasing knowledge of CPE action at the molecular level is not only permitting development of several translational applications, as described in the preceding section, but is also suggesting new approaches to control CPE-mediated intestinal disease. For example, synthetic peptides corresponding to the CPE binding region (ECL-2) of the claudin 4 receptor have been shown to specifically interfere with CPE action in rabbit small intestinal loops [90] (Figure 5). Such agents might prove useful as therapeutics for treating exceptionally severe cases of CPE-mediated food poisoning or CPE-mediated non-food-borne diseases, which can be chronic (lasting for multiple days to weeks) and are often more severe than CPE-mediated food poisoning. CPE also represents an exquisite, increasingly used tool for probing tight junction structure and function.
